# An arbuscular mycorrhiza from the 407‐million‐year‐old Windyfield Chert identified through advanced fluorescence and Raman imaging

**DOI:** 10.1111/nph.70655

**Published:** 2025-11-12

**Authors:** Christine Strullu‐Derrien, Raymond Wightman, Liam Patrick McDonnell, Gareth Evans, Frédéric A. Fercoq, Paul Kenrick, Andrea C. Ferrari, Sebastian Schornack

**Affiliations:** ^1^ Science Group The Natural History Museum Cromwell Road London SW7 5BD UK; ^2^ Institut Systématique Évolution Biodiversité (UMR 7205), Muséum National d'Histoire Naturelle, CNRS, Sorbonne Université, EPHE, UA 75005 Paris France; ^3^ Sainsbury Laboratory University of Cambridge Cambridge CB2 1LR UK; ^4^ Cambridge Graphene Centre University of Cambridge Cambridge CB3 0FA UK; ^5^ Unité Molécules de Communication et Adaptation des Micro‐organismes (MCAM, UMR7245), Muséum National d'Histoire Naturelle, CNRS 75005 Paris France

**Keywords:** AM mycorrhizas, confocal scanning laser microscopy, Devonian, fluorescence lifetime imaging microscopy, fossil record, plant symbioic fingus, Raman Spectroscopy, 3D imaging

## Abstract

- Mycorrhizal associations between fungi and plants are a fundamental aspect of terrestrial ecosystems. Mycorrhizas occur in *c*. 85% of extant plants, yet their geological record remains sparse. Rare fossil evidence from early terrestrial environments offers crucial insights into these ancient symbioses, but visualizing fossil fungi at the microscale within plant tissues is challenging.
- Here, we combine confocal laser scanning microscopy and fluorescence lifetime imaging microscopy (FLIM) to investigate a newly identified fungus and cellular structures of a 407‐Myr‐old plant from the Windyfield Chert, a stratigraphically distinct fossiliferous unit from Rhynie (Scotland). We also applied Raman spectroscopy to investigate the carbon framework of both fungal and plant tissues.
- This integrative approach revealed fungal structures in unprecedented detail. The fungus, *Rugososporomyces lavoisierae* gen. nov., sp. nov., exhibits features resembling extant *Glomeromycotina* arbuscular mycorrhizal fungi. This is the first record of mycorrhizas from the Windyfield Chert. FLIM further distinguished features at the subcellular level, while Raman spectroscopy showed that fungal arbuscules and vesicles of the plant water‐conducting cells underwent geological alterations, resulting in a similar chemical composition.
- These findings expand our understanding of ancient and extremely rare plant–fungal symbioses and highlight the potential of confocal‐FLIM for advancing palaeobotanical research.

## Abstract (fr)

- Les associations mycorhiziennes entre les plantes et les champignons sont omniprésentes dans les écosystèmes terrestres actuels. En effet, elles concernent ~85% des plantes, mais leur histoire évolutive reste peu connue. De rares traces fossiles trouvées dans les premiers environnements terrestres offrent cependant des informations capitales. Il est toutefois très difficile de visualiser ces structures symbiotiques à l’échelle microscopique.
- Dans cet article nous avons utilisé la microscopie confocale laser associée à la *microscopie de temps de vie de fluorescence (FLIM)* pour étudier un nouveau champignon et les structures cellulaires d’une plante du chert de Windyfield datant de 407 million d’années. Ce niveau géologique est distinct du chert de Rhynie, l’autre niveau fossilifère situé dans le village de Rhynie en Ecosse. Nous avons aussi utilisé la spectroscopie Raman pour examiner la composition carbonée des tissus du champignon et de la plante.
- Cette approche intégrée a permis de révéler les structures fongiques avec des détails jamais obtenus jusqu’alors. Le champignon *Rugosomycetes lavoisieriae* gen. nov., sp. nov. possède des caractères similaires à ceux des champignons mycorhiziens actuels du groupe des *Glomeromycotina.* C’est la première fois que l’on observe des mycorhizes dans le chert de Windyfield. La technique FLIM a permis d’observer des détails au niveau subcellulaire. La spectroscopie Raman, elle, a montré que les arbuscules fongiques et les vésicules du système conducteur de la plante avaient subi des altérations géologiques, et avaient maintenant la même composition chimique.
- Ces résultats permettent de mieux comprendre ces symbioses anciennes, et extrêmement rares, entre la plante et le champignon. Ils montrent aussi le potentiel de l’approche combinée Confocal‐FLIM pour les recherches en paléobotanique.

## Abstract (es)

- Las asociaciones micorrízicas entre hongos y plantas son fundamentales para los ecosistemas terrestres. Aunque las micorrizas están presentes en aproximadamente el 85 % de las plantas existentes, sus registros geológicos siguen siendo escasos. La escasa evidencia fósil existente de los primeros ambientes terrestres ofrece información crucial sobre estas antiguas simbiosis. Sin embargo, es todo un reto visualizar fósiles de hongos dentro de los tejidos vegetales a las pequeñas escalas requeridas.
- En este estudio se combinan la microscopía confocal láser de barrido y la microscopía de imágenes de tiempo de vida de fluorescencia (FLIM) para investigar la asociación micorrízica entre una nueva especie de hongo y las estructuras celulares en una planta de 407 millones de años de antigüedad. Las muestras proceden del Windyfield Chert, una unidad estratigráfica fosilífera definida situada en Rhynie (Escocia). También se utilizó la espectroscopia Raman para investigar la estructura de carbono de los tejidos fúngicos y vegetales.
- Este enfoque integrado reveló estructuras fúngicas con un nivel de detalle sin precedentes. El hongo, *Rugososporomyces lavoisieriae* gen. nov., sp. nov., presenta características similares a las de los hongos micorrízicos arbusculares *Glomeromycotina* ahora existentes. Este es el primer registro de micorrizas en el Windyfield Chert. La FLIM fue capaz de identificar características a nivel subcelular, mientras que la espectroscopia Raman mostró que los arbúsculos fúngicos y las vesículas de las células conductoras de agua de las plantas fueron alteradas geologicamente, lo que resultó en una composicion química similar de ambas.
- Estos hallazgos únicos amplían nuestra comprensión de simbiosis entre plantas y hongos my antiguas y muy infrecuentes, a la vez que resaltan el potencial de la combinación de las técnicas Confocal‐FLIM para el avance de la investigación paleobotánica.

## Introduction

Fungi and plants have a lengthy record, dating back to the early Devonian, and they have interacted with each other in diverse ways for much of the history of life on land (Berbee *et al*., 2020). The fossil record provides rare insights into the evolution of fungi as symbionts and pathogens of plants as well as crucial evidence of their roles as saprotrophs (e.g. Krings *et al*., 2007; Taylor *et al*., 2015; Strullu‐Derrien *et al*., 2019a). The oldest geological evidence for the endomycorrhizal symbiosis comes from the 407‐Myr‐old Rhynie Chert in Scotland (UK) (Edwards *et al*., 2018; Strullu‐Derrien *et al*., 2019a). The cherts formed as hydrothermal waters rich in silica spread across a wetland community, entombing the organisms and preserving them in exquisite detail. The fossils within the cherts are studied typically in petrographic thin section using brightfield microscopy (Taylor *et al*., 2015; Strullu‐Derrien *et al*., 2019a). Despite their frequently excellent preservation at Rhynie, fungi and other microorganisms are challenging to document due to their small size and the limitations of brightfield microscopy. Confocal laser scanning microscopy (CLSM) has previously been applied to the fungal (Strullu‐Derrien *et al*., 2016, 2018, 2023a), algal (Strullu‐Derrien *et al*., 2021), protist (Strullu‐Derrien *et al*., 2019b) and cyanobacterial (Strullu‐Derrien *et al*., 2023b; McMahon *et al*., 2024) components of this system to bring structures of a few micrometres into sharper focus through optical sectioning, and to reconstruct organisms and their interactions in three dimensions. CSLM surpasses brightfield microscopy by suppressing image blurring caused by out‐of‐focus light. As it relies on autofluorescence, it enhances contrast between organics and the mineral matrix. This enables high‐resolution, three‐dimensional imaging of fossils and digital reconstruction of intricate cellular and subcellular structures without physical sectioning.

The possibility that Rhynie plants hosted endomycorrhizal fungi was first suggested by Kidston & Lang (1921) in their original descriptions of the fossil fungi. This idea was later developed by Boullard & Lemoigne (1971). Contrary to preconceived ideas, arbuscular mycorrhizas (AM) are rarely observed in the fossil plants at Rhynie, and arbuscules, the hallmark of AM, have only been documented in two plants. The first compelling evidence for arbuscules came from the sporophyte of *Aglaophyton majus* in the Rhynie Chert Unit (Remy *et al*., 1994) in the form of a fungus attributed to *Glomeromycota* (Taylor *et al*., 1995), which was later also noted in its young gametophyte (Taylor *et al*., 2005). Another similar fungus was reported in *Nothia aphylla* (Krings *et al*., 2007), but no arbuscules were observed. Further evidence of intracellular coils and arbuscules was reported in the plant *Horneophyton lignieri* from the same stratigraphic unit (Strullu‐Derrien *et al*., 2014). Two distinctive types of fungus were observed within different tissues of this plant, indicating simultaneous colonization by species attributable to both *Glomeromycotina* and *Mucoromycotina* (Strullu‐Derrien *et al*., 2014).

Here, we combined CLSM with fluorescence lifetime imaging (FLIM) to investigate the fine structure of a newly identified arbuscular mycorrhizal fungus observed within the aerial axis of the early land plant *Aglaophyton majus*. The material comes from the Windyfield Chert, a stratigraphically defined unit distinct from the Rhynie Chert unit but not considered to be significantly different in age (Wellman, 2006). At Windyfield, palaeoenvironments ranged from terrestrial laminated, brecciated and vegetated sinter sheets to low‐temperature pools and marginal aquatic settings. Fluid deposition at Windyfield took place closer to the centre of hydrothermal activity and cherts exhibit more brecciation. Overall, the cherts at Windyfield display a higher content of detrital material (Fayers & Trewin, 2004). Fayers & Trewin (2004) found the biota to be comparable in both chert units, but there is a greater richness of arthropods at Windyfield, and there is one species of plant unique to each site (Powell *et al*., 2000; Trewin & Kerp, 2018). Recent work has shown that there are also some differences in terms of animal (nematodes), protist, fungal and cyanobacterial components (Poinar *et al*., 2008; Strullu‐Derrien *et al*., 2019b, 2023a). While several fungi have been described from the Windyfield chert (e.g. Krings & Harper, 2017; Krings, 2022), it was unknown whether the environmental conditions of the Windyfield system would enable plants to engage with symbiotic fungi.

Unlike traditional fluorescence microscopy, which creates an image based on fluorescence emission intensity (Lichtman & Conchello, 2005), FLIM is a technique that measures the time a fluorophore remains in an excited state before emitting a photon (Datta *et al*., 2020) and thus enables further discrimination of structures by measuring the decay rate of the fluorescence, which can reflect differences in underlying chemistry. Confocal‐FLIM uses a pulsed laser and time‐correlated single photon counting detection that give an output of fluorescence decay, with a fast decay indicating short lifetime components and a slow decay indicating long lifetimes (Datta *et al*., 2020; Monteleone *et al*., 2021). Lifetime changes are sensitive to the immediate changes in the environment of the fluorophore and its composition (Monteleone *et al*., 2021; Ali & Kundu, 2025). This sensitivity was demonstrated in living plants, with changes in fluorescence lifetime of lignin, measured by confocal‐FLIM, corresponding to small changes in the chemical structure of lignin, measured by Raman microscopy (Wightman *et al*., 2019; Escamez *et al*., 2021). Considering the high sensitivity (measured with temporal resolution of picoseconds) of FLIM to the chemical environment, here we use fluorescence lifetime in a novel way to image fossilized fungal and plant structures. Raman spectrometry is then employed to investigate the carbon framework of the fossils. This is a nondestructive approach enabling the identification of specific organic moieties and providing insight into the fossil's geological history. This integrative approach is promising for distinguishing among chemically different organic and mineral backgrounds, which is not possible with conventional light microscopy.

## Materials and Methods

The fossiliferous chert deposits at Rhynie are located in an outlier of Lower Old Red Sandstone situated *c*. 50 km north‐west of Aberdeen, UK (Trewin, 2004). Here, there are two distinct chert units that developed from their own centres of surface hydrothermal activity with hot springs and geysers. The materials described here come from the Windyfield Cherts Unit, a stratigraphically fossiliferous deposit discovered in 1988 as float blocks, *c*. 700 m from the original Rhynie Chert site (Trewin & Kerp, 2018). Trenching at Windyfield in 1997 revealed pods of chert *in situ* interbedded with fluvial and lacustrine sandstones and hydrothermally altered shales (Fayers & Trewin, 2004). The Windyfield Chert Unit is within the Dryden Flags Formation, lying *c*. 550 m above the Rhynie Chert Unit (Rice & Ashcroft, 2004). Very little material from Windyfield is available for study, and there are far fewer prepared thin sections.

Sinter deposition of both chert units originally took place in an intermontane basin on the southern margin of the palaeocontinent of Laurussia, with sediment accumulating on a low‐energy alluvial plain (Rice & Ashcroft, 2004). Silicification ensued from geothermal outwash of alkali‐chloride hot springs at some distance from vents and at different temperatures (Fayers & Trewin, 2004). Plants were silicified close to their sites of growth and occasionally in growth position, together with their substrates.

A series of thin sections was prepared by Callum Hatch at the Natural History Museum, London, using the petrographic standard method. They were cut from a chert block owned by the National Museum of Scotland, Edinburgh. Sections are *c*. 70 μm in thickness; they are mounted without glass coverslips, but the rock surface is highly polished. The results presented here are from slide no. NMS G.2022.11.48.1. The specimens (*Rugososporomyces lavoisierae* Strullu‐Derrien and Schornack gen. nov., sp. nov.) were studied with a Nikon Eclipse LV100ND compound microscope at the Natural History Museum; depth of field was enhanced through z‐stack montage. Digital light microscopy was also carried out on a Keyence VHX7000 at the Sainsbury Laboratory, Cambridge, with overview images made up from merged tile scans.

For the analyses in CLSM, preliminary overviews and imaging of the vascular strand and fungal spore were done at the MNHN light microscopy facility (CeMIM, Paris) on a Zeiss LSM880 confocal microscope using a 40× 1.30 NA Plan Apo oil immersion objective lens. Samples were excited with the 561 nm laser, and the autofluorescence signal was collected with either the Airyscan head using a 32 GaAsP detector array in super resolution mode or with the 32 channel GaAsP spectral detector. Images were recorded with pixel dimensions of 50–70 nm and 16‐bit depth mode. For, imaging the vascular strand, a 13 μm z‐stack was acquired with steps of 0.5 μm. Images were processed in Zen Black software (Carl Zeiss) to deconvolve Airyscan images (Airyscan processing) and spectral images (Linear unmixing). For linear unmixing, the main autofluorescence spectrum was extracted using the ‘Auto Find’ option in Zen Black.

Subsequent CLSM imaging at the Sainsbury Laboratory, Cambridge, used a Leica SP8 X upright confocal microscope with a 561 nm laser and a HyD detector with an emission window of 633–791 nm. A 63× 1.4 NA oil immersion objective lens was used. Z stacks were acquired using the software optimized z‐spacing.

Fluorescence Lifetime Imaging microscopy (FLIM) was performed on a Leica SP8‐SMD upright confocal microscope equipped with Picoquant FLIM hardware running both LAS X and SymphoTime software. The excitation source was a 440 nm pulsed laser operating at 20 MHz. The sample was viewed using a 63× 1.4 NA oil immersion objective. Detection of the signal used an SMD‐HyD detector set to an emission window between 601 and 786 nm, photon counting mode. The laser power was altered to achieve a max count rate of 2000 cps. FLIM settings on the LAS X were 848 × 848‐pixel format, unidirectional scanning, zoom 1.75, and with 70 FLIM iterations. Z stacks were acquired using LAS X software‐optimized z‐spacing. Average lifetime maps were generated using the FastFLIM calculations and displayed on SymphoTime. Curve fitting and extraction of images representing the discrete lifetimes were carried out on an offline SymphoTime workstation. The data follows a triple exponential decay with a very short (picosecond) range lifetime representing fungal structures (or vesicles of the conducting tissue) only and a long nanosecond lifetime representing fungus plus plant (or vesicles of the confocal tissue plus other plant tissues). An intermediate low nanosecond lifetime represents a low intensity background.

Raman spectra were acquired using Renishaw Invia and Horiba HR800 spectrometers using excitation wavelengths of 325, 457, 488, 514, 532 and 633 nm. For all wavelengths, the incident power was limited to < 1 mW to avoid both local heating and damage to the sample. The spectral scan range was 0–3400 cm^−1^. Most spectra were measured using a ×100 objective with a Numerical Aperture (NA) = 0.95, resulting in a laser spot size of *c*. 1 μm. The 325 nm measurements used a ×40 objective suitable for Near Ultraviolet (NUV) excitation. At each wavelength, the spectra were calibrated using the Si Raman peak at 520.7 cm^−1^ (Temple & Hathaway, 1973). Spectra were also acquired away from the regions of interest (ROIs) to remove background scatter and luminescence during baseline correction.

Multiple Raman spectra ROIs were chosen based on the FLIM: (1) dark structures in the fungal zone, corresponding to the short (picosecond) fluorescence lifetime of the fungal arbuscules; (2) vesicles of the conducting tissue at the center of the plant axis, again giving the short lifetime. Supporting Information Fig. S1 shows these regions with an overlay of a Raman map of the G‐band peak that was sampled at 10‐micron intervals from the periphery to the center of the plant axis. This was acquired with the 514 nm laser and a 20x oil immersion objective lens on a Renishaw InVia Raman microscope in mapping acquisition mode and a static scan with a 2400 l mm^−1^ grating centered at 1400 cm^−1^. Signal corresponding to observable G‐bands is found in the fungal and vascular strand regions, but was not observed between the fungal region and the periphery of the axis, which corresponds to a region devoid of fungal colonization.

The nature of the fossilization process at Rhynie involving silicification does not enable the use of staining methods that have been applied to other fossil and extant specimens in which chitin or chitin‐like residues exist in different permineralization backgrounds (e.g. dolomitic shale, Bonneville *et al*., 2020).

## Results

**Fig. 1:**
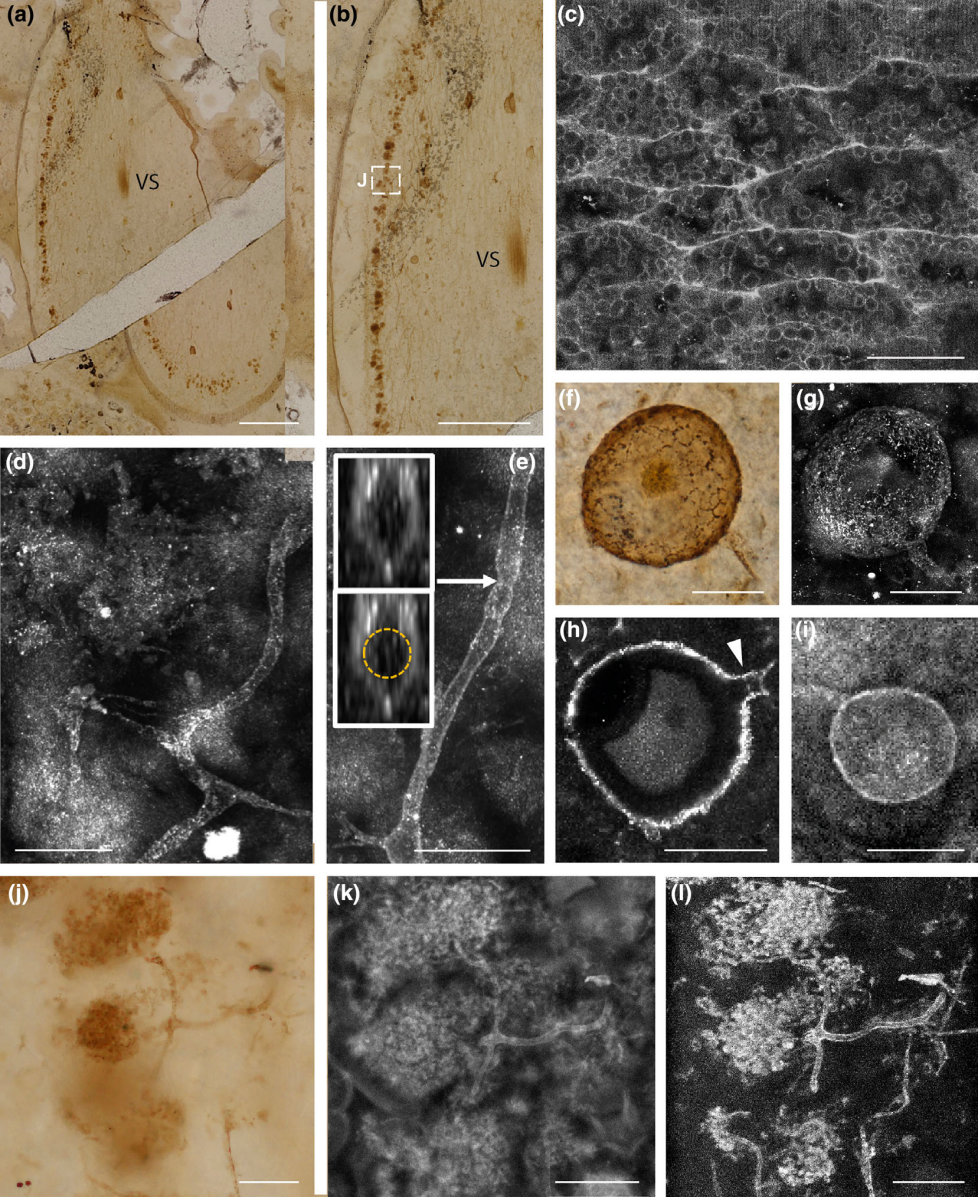
Arbuscular mycorrhizas in *Aglaophyton majus* and comparative microscopy. (a, b) Slightly oblique transverse section of the plant axis showing the fungal arbuscular zone at the periphery of the inner cortex (white box) and the central strand of water‐conducting cells (brightfield microscopy). (c) Water‐conducting cells of the vascular strand showing the vesicular structure (CLSM spectral). (d) H branching in hyphal development (CLSM). (e) Hypha with pseudoseptum. Sections through the hypha (insets) show that there is no internal septum (CLSM). (f) Spore showing rugose surface (brightfield microscopy). (g) Maximum projection image of a fungal spore (CLSM). (h) Single orthoslice image showing the structure of the wall and the septum on the branching hypha (arrow) (CSLM & Airyscan). (i) Vesicle and branching hypha (CLSM). (j–l) Comparative microscopy: arbuscules in brightfield microscopy (j), CLSM (k) and FLIM – short (0.3 ns) lifetime (l). Bars, 0.8 mm (a, b); 30 μm (c–l). Slide no NMS G.2022.11.48.1. See also the original datasets https://doi.org/10.5281/zenodo.15194427 (Strullu‐Derrien *et al*., 2025).

We report the identification of fossil structures representing an AM fungus with distinct features (Fig. 1). *Rugososporomyces lavoisierae* Strullu‐Derrien & Schornack *sp. nov*. was found in axes of the plant *Aglaophyton majus* within the Windyfield Chert Unit, dated to the Lower Devonian (Mark *et al*., 2011).

### FLIM distinguishes fungal from plant structures

**Fig. 2:**
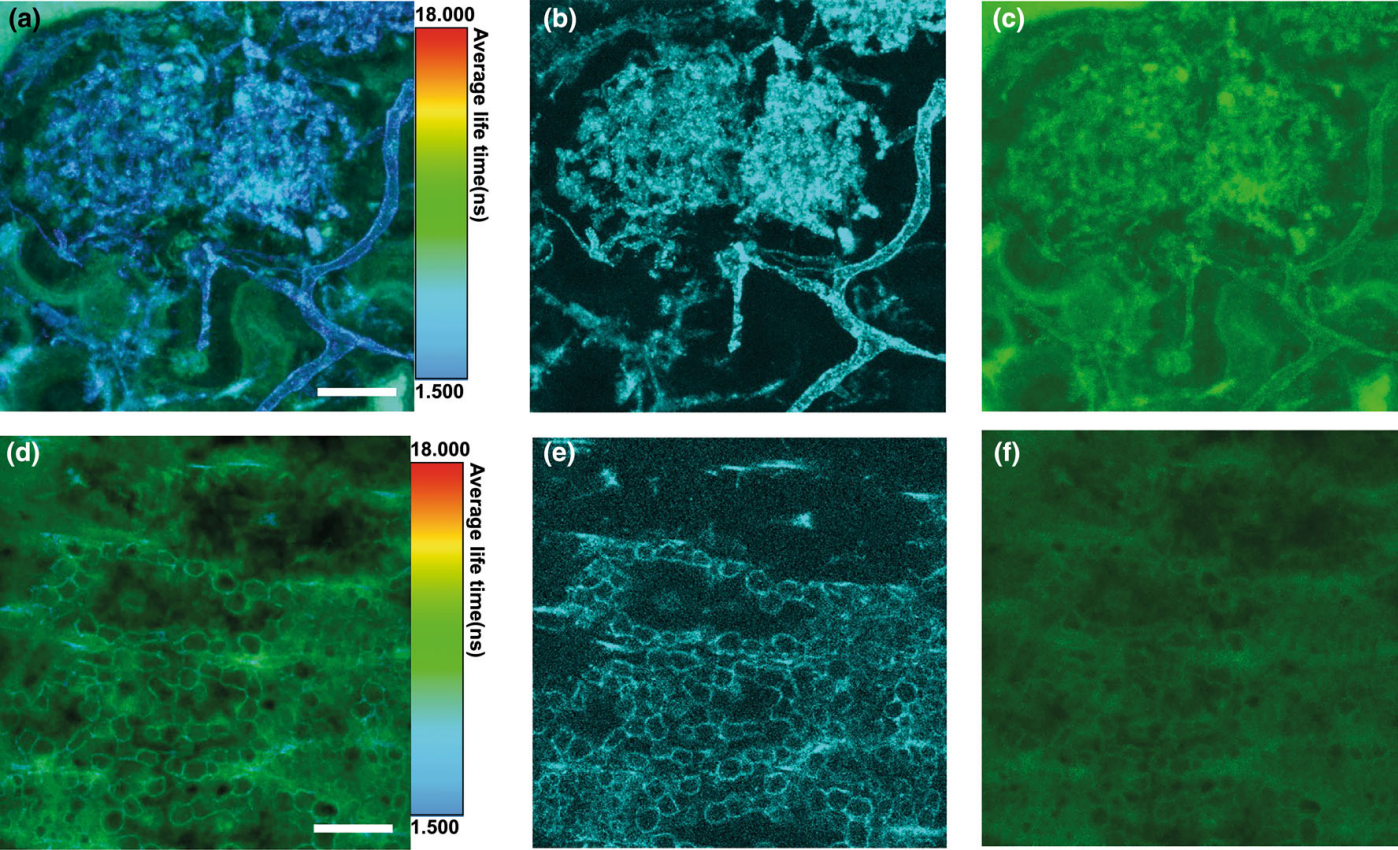
FLIM analyses of the fungal and plant structures. (a) Maximum projection of a 23 slice z‐stack showing a relatively short average lifetime of autofluorescence of the arbuscules (blue/cyan) compared to areas of the plant devoid of fungal structures (green). (b) Maximum projection of the very short (picosecond) lifetime that represents the arbuscules. (c) Maximum projection of the long lifetime representing plant‐plus‐arbuscules. (d) Single optical section through the central strand of the aerial axis of *Aglaophyton majus* showing the average lifetime of autofluorescence. Same optical section showing the very short (picosecond) lifetime (e) and long (nanosecond) lifetime (f). The short lifetime gives high signal in the ovoid structures (vesicles) within the water‐conducting cells. Bars, 20 μm. Slide no NMS G.2022.11.48.1. See also the original datasets https://doi.org/10.5281/zenodo.15194427 (Strullu‐Derrien *et al*., 2025).

We imaged both fungus and tissues of the host plant using brightfield microscopy (Fig. 1a,b,f,j), CLSM (Fig. 1c–e,g–i,k,l) and fluorescence lifetime imaging microscopy (FLIM) (Fig. 2). CLSM reveals key features of the hyphae (lack of septum, Fig. 1e), fungal spores (detail of the spore wall and subtending hypha, Fig. 1g,h), vesicles (Fig. 1i) and arbuscules (Fig. 1j–l). CLSM‐based imaging outperforms brightfield microscopy by improving image clarity (Figs 1j vs 1k). For plant tissues, CLSM reveals the vesicular structure of the water‐conducting cells of the host plant *A. majus*, a well‐preserved feature (Fig. 1c; Notes S1; Fig. S2A), which is diagnostic of this tissue (Remy & Hass, 1996; Edwards, 2003).

FLIM of a fungal arbuscule located in a plant cell returns average fluorescence lifetimes that emphasize different features of the plant and fungal structures (Fig. 2a–c). A short average fluorescence lifetime of 0.3 ns separates the fungus (arbuscule and other hyphae) from plant cell structures as well as any other background (Fig. 2b). Longer average lifetimes > 6 ns give signals from both fungus and surrounding structures (Fig. 2c). Combining both shorter and longer lifetimes highlights where the plant cell contributes uniquely to the longer fluorescence lifetime (Fig. 2a). Similarly, FLIM of the plant water‐conducting cells, but without obvious fungi, returns average fluorescence lifetimes that pick out different features of the specimen (Fig. 2d–f). The short 0.3 ns lifetime gives a clear signal from the vesicles of the conducting tissue (Fig. 2e). The longer > 6 ns lifetime provides a more uniform signal of lower intensity from the plant, and there is little structure in the image (Fig. 2f).

Compared to other light microscopy methods, the imaging of fungus and host plant using CLSM gives better definition of cell structures due to the improved axial resolution that is inherent to CLSM. Combining CLSM with FLIM provides extra information by separating features based on fluorescence lifetime (Video S1). By observing just the short 0.3 ns fluorescence lifetime, a clearer image can be obtained for both fungal arbuscules and vesicles of the plant water‐conducting cells. This yields a greatly improved signal‐to‐noise ratio, and the residual organic‐derived carbon in the fossil is the source of this short‐lifetime fluorescence.

### Raman investigation of the fluorescent carbonaceous material of the fungal and plant cell walls

**Fig. 3:**
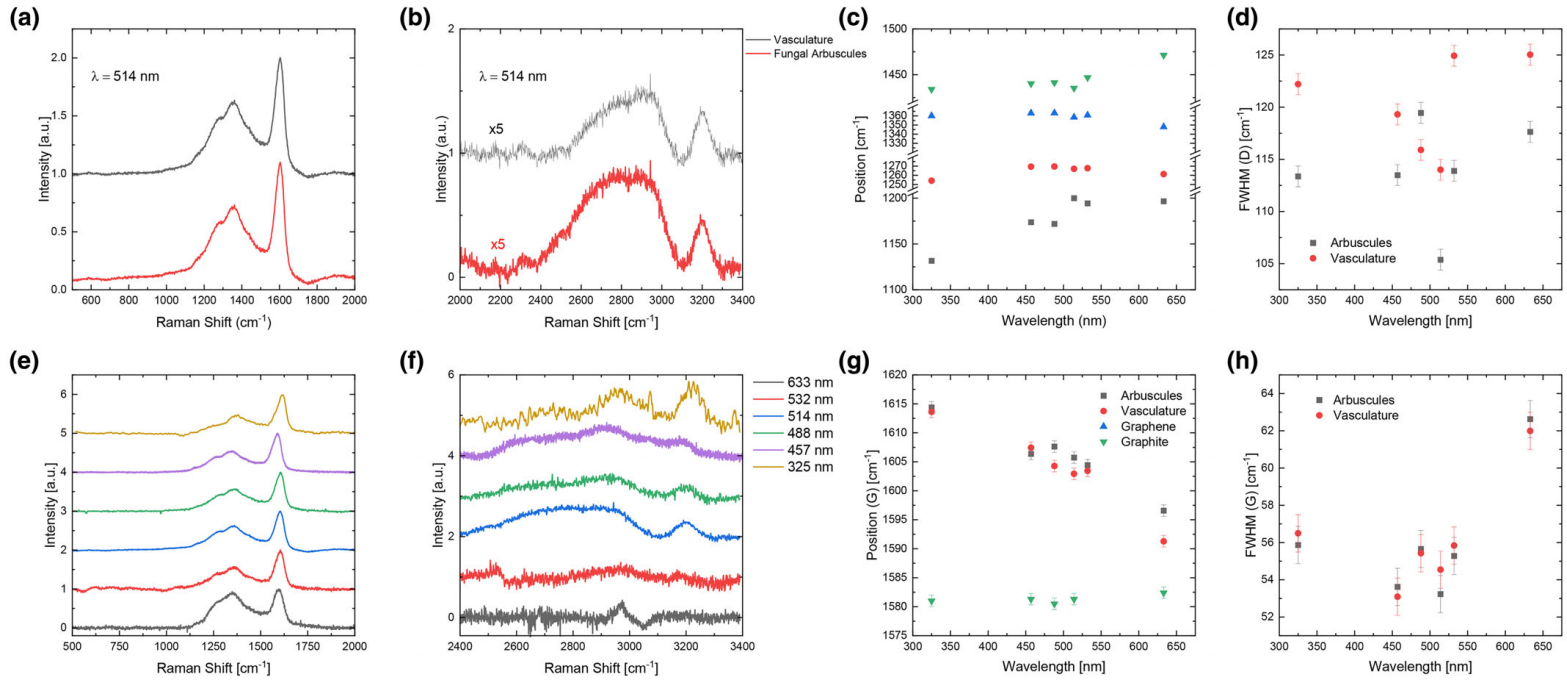
Raman analyses of the fungal and vasculature structures. (a, b) Raman spectra for the vesicles of the water conducting cells (black) and arbuscules (red) measured at 514 nm. (e, f) Raman spectra acquired at multiple wavelengths for the arbuscules. (c, g) Pos (d), Pos (g) and of subbands *c*. 1266 and 1450 cm^−1^ at 514 nm, as a function of wavelength. (d, h) FWHM (g) for both fungal arbuscules and vesicles of the water conducting cells.

Both fungal arbuscules and vesicles of the plant conducting cells displayed similar FLIM signatures, which differed from surrounding plant cells and background. To probe the nature of the organic signal underpinning the FLIM results in Fig. 2, the arbuscules of the fungus and the vesicles of the water‐conducting cells in the vascular strand of the host plant were analyzed using Raman spectroscopy (Fig. 3a–h).

Raman spectra were taken for both the fungal arbuscules and the vesicles of the water‐conducting cells, as shown in Fig. 3a,b. For both samples, the spectra are characterized by *c*. 4 peaks between 1000 and 1650 cm^−1^. The spectra acquired at 325, 457 and 488 nm also show several peaks between 2800 and 3200 cm^−1^ in both samples (Fig. 3e,f). Peaks *c*. 1358 and 1608 cm^−1^ are assigned to the D and G peaks, respectively, and arise due to the presence of sp^2^ carbon (Ferrari & Robertson, 2001a; Ferrari *et* al., 2006). The UV spectra in Fig. 3f have peaks *c*. 2960 and 3220 cm^−1^, assigned to C–H stretching and to the 2D’, respectively (Casiraghi *et al*., 2005). Analysis of the D and G peaks including their positions, Full Width at Half Maximum (FWHM) (Fig. 3d,h), intensity ratios, dispersion derived from the peak positions in Fig. 3c,g and henceforth referred to as Disp (i.e. the shift in frequency as a function of excitation energy) can be used to determine the nature of the underlying sp^2^ carbon. FWHM(G) for both fungal arbuscules and vesicles of the water‐conducting tissue is *c*. 56 cm^−1^ at 514 nm, consistent with nanocrystalline sp^2^ domains (Ferrari & Robertson, 2001a; Ferrari *et al*., 2003). The peaks *c*. 1266 and 1433 cm^−1^ (at 514 nm) are consistent with those found in carbon chains (Ferrari & Robertson, 2001a; Ehrenfreund *et al*., 1987; Ferrari & Robertson, 2004), possibly derived from the transformation of precursor compounds in the arbuscules and vesicles of the water‐conducting cells such as chitin or cellulose.

For the fungal arbuscules as well as the vesicles of the water‐conducting cells, Disp(D) is *c*. 4.1 (8.2) cm^−1^/eV and Disp(G) is *c*. 8.3 (10.3) cm^−1^/eV, consistent with both regions containing similar sp^2^ carbon (Ferrari & Robertson, 2001a, 2001b). The much lower Disp(D) with respect to graphite (Ferrari & Robertson, 2001a, 2001b) means that sp^2^ domains only span a limited distribution in sizes and do not combine to form a long‐range graphitic lattice.

The intensity ratio of the D and G peaks, I(D)/I(G), can be used to estimate the mean size L_a_ of the sp^2^ clusters (Tuinstra & Koenig, 1970; Ferrari & Robertson, 2000). For the fungal arbuscules and vesicles of the water‐conducting cells, we have I(D)/I(G) *c*. 0.47–0.6 at λ = 514 nm, implying a mean length of the sp^2^ clusters *c*. 1.1–1.2 nm (Ferrari & Robertson, 2000; Cançado *et al*., 2011; Bruna *et al*., 2014). These data suggest that the investigated fossilized fungal and plant material has undergone diagenetic alteration, resulting in the formation of nanocrystalline carbon structures.

### A morphologically distinct endomycorrhizal fungus colonizing the plant *Aglaophyton majus*

#### Systematics

Kingdom – *Fungi* R.T. Moore.

Phylum – *Mucoromycota* Doweld, emend. Spatafora and Stajich.

Subphylum – *Glomeromycotina* (C. Walker and A. Schüßler) Spatafora and Stajich, subphylum and *stat. nov*.

Class – *Glomeromycetes* Caval.‐Sm.

Order – *Incertae sedis –* Strullu‐Derrien & Schornack.

Genus – *Rugososporomyces* Strullu‐Derrien & Schornack gen. nov.

Etymology – *Rugoso* refers to the nature of the spore wall; *sporo* is from the Greek *spora* meaning spore; *myces* is the Latin word for fungus.

Genus diagnosis – Fungus with aseptate intercellular hyphae with H branching, spores, vesicles and intracellular arbuscules. Differs from other fungi in the Rhynie Chert in the following ways: spore wall possibly bilayered, with a relatively thick, rugose wall; smaller diameter of the branching hyphae; hyphae terminating in a spore are either smaller or larger than other hyphae; the ratio of spore diameter to hyphal width is smaller; the spores are comparatively either smaller or larger.

Species – *Rugososporomyces lavoisierae* Strullu‐Derrien and Schornack sp. nov.

Etymology – In honor of Marie‐Anne Paulze de Lavoisier (1758–1836), who was a collaborator of her husband, Antoine Laurent de Lavoisier and was his laboratory assistant. The scientific collaboration of this husband‐wife team is perhaps unique among the giants of respiratory physiology (West, 2013). Together, they pioneered Physiology and established the basis of modern Chemistry, two areas relevant to our study.

Species diagnosis – Branched hyphae 3.5–6.5 μm in diameter; unbranched hyphae 7 μm in diameter when terminating in a spore and 4 μm in diameter when terminating in a vesicle; basal stalk of the arbuscules 3.5 μm in diameter. Spores globose, up to 74 μm in diameter, with a 3.1 μm thick rugose and possibly bilayered wall. Subtending hypha closed by a septum. Vesicles range from globose (up to 39 μm in diameter) to elongate (41 μm wide, 50 μm long).

Holotype – specimens in slide no. NMS G.2022.11.48.1 at the National Museum of Scotland, Edinburgh. Fig. 1.

Locality – Rhynie, North‐West of Aberdeen (Scotland): Windyfield Cherts Unit (Rice & Ashcroft, 2004).

Age – Lower Devonian (407.1 ± 2.2 Ma) (Mark *et al*., 2011).

Mycobank (Crous *et al*., 2004; Robert *et al*., 2013) nos: MB 860889 (genus); MB 860890 (species).

#### Description of fungus and its known distribution in the plant

*Rugososporomyces lavoisierae* was observed in an oblique transverse section of the axis of the fossil plant *Aglaophyton majus* (Fig. 1a,b). The outlines of epidermal cells are preserved in the cuticle. Fungal colonization occurs in a circumferential continuous zone (Fig. 1a). Fungal structures such as hyphae, spores, vesicles and intracellular arbuscules differ in their relative abundance in the different zones of the plant axis. Part of the inner cortex is dominated by intercellular hyphae and surrounded by a zone in which arbuscules develop within turgescent cells (see Fig. S2B), and which also contains hyphae, vesicles and spores; the outer cortex is devoid of fungal colonization (Fig. 1b). Hyphae form H branching (Fig. 1d). Detailed investigation of the hyphae in 3D reveals pseudo‐septa that seem to be bends in the hyphal wall rather than true septa (Fig. 1e), thus all hyphae are aseptate except for the point of attachment of the spore in which the hyphal lumen is closed by a septum (Fig. 1h). Hyphae are variable in width: branched forms range from 3.5 μm to 6.5 μm in diameter; unbranched forms are 7 μm in diameter when terminating in a spore and 4 μm in diameter when terminating in a vesicle (Fig. 1f–i). Spores are globose (up to 74 μm in diameter) and possibly bilayered, although layers are not easily discernible (Fig. 1h), with a thickened (3.1 μm thick) rugose wall (Fig. 1f–h). Vesicles, corresponding to hyphal swellings, are of varying shape from globose (up to 39 μm in diameter) to elongate (41 μm wide, 50 μm long) (Fig. 1i). Intracellular arbuscules develop at the top of branched hyphae that are 3.5 μm in diameter (Fig. 1j–l). Hyphae of this type have not been observed in proximity to and external to the plant axis, so we were unable to illustrate the point of penetration.

#### Comparison with endomycorrhizal G*lomeromycotina* at Rhynie

*Rugososporomyces lavoisierae* differs from other endomycorrhizal *Glomeromycotina* at Rhynie primarily in its spores, vesicles and its ratio of spore to subtending hyphal diameter (Table 1). *Rugososporomyces lavoisieriae* can be distinguished from *Glomites rhyniensis* (Taylor *et al*., 1995) primarily on the size and structure of the spores and vesicles and on the dimensions attained by hyphae in the inner cortex. The hyphae of both species are aseptate; occasional septation of narrow, thin‐walled hyphae was noted in *G. rhyniensis*. However, these might be a taphonomic artifact like the pseudoseptae that we observed during confocal microscopy imaging in *R. lavoisierae* (Fig. 1e). H branching is a feature of the hyphae in both species, but the hyphae in the inner cortex of *R. lavoisierae* are much narrower (3.5–6.5 μm) than those of *G. rhyniensis* (8–14 μm). The spores of *G. rhyniensis* have a smooth, thick (6 μm), multilayered wall with an inner layer that is continuous with the subtending hypha, whereas the spores of *R. lavoisierae* have a thinner (3.1 μm), rugose, possibly bilayered wall with a septum on the subtending hypha. The spores of *G. rhyniensis* are globose to elongate and 50–80 μm in diameter, whereas those of *R. lavoisierae* are more globose but of similar maximum size (*c*. 74 μm). However, the spore category of *G. rhyniensis* also encompasses vesicles, which in *R. lavoisierae* are elongate or globose but much smaller structures (41 × 50 μm; up to 39 μm in diameter). The arbuscules of *G. rhyniensis* and *R. lavoisierae* are indistinguishable: both developed from narrow hyphae (*c*. 3.5 μm wide) giving rise to 2–3 major branches that led to clusters of much branched narrower hyphae.

**Table 1:** Comparison of the key features of *Rugososporomyces lavoisierae* and other endomycorrhizal *Glomeromycotina* at Rhynie. Slide no NMS G.2022.11.48.1.

Fungus	Host	Coloniza‐tion	Hyphae	Spore diameter	Arbuscules	Vesicle size	Fungal entry	References
*Glomites rhyniensis*	*Aglaophyton majus* Rhynie Chert	Aerial axis and rhizome	Aseptate branched hyphae 5–18 μm in diam. Hyphae 5 μm attached to spores	Globose‐elongate Multilayered spores 50–80 μm No septa Thickness of the wall: 6 μm	Present	?	Entry between the rhizoids, by stomata	Remy *et al*. (1994), Taylor *et al*. (1995)
*Rugososporomyces lavoisierae*	*Aglaophyton majus* Windyfield chert	Aerial axis	Aseptate branched hyphae 3.5 to 6.5 μm Hyphae 7 μm attached to spores, 4 μm attached to vesicles	Globose Possibly bilayered, rugose, relatively thick‐walled spores Up to 74 μm Septa on the branching hypha Thickeness of the wall: 3.1 μm	Present	Globose, up to 39 μm or elongate (41 μm wide, 50 μm long)	?	This paper
Not named	*Aglaophyton majus*, *Rhynia gwynne‐vaughanii* Rhynie Chert	Aerial axis and rhizome	Aseptate hyphae. Hyphae 5.5 to 7 μm attached to spores	Elongate globose spores 39 × 45 μm	?	?	?	Boullard & Lemoigne (1971)
Fungus n°3 ressembling *Glomites rhyniensis*	*Nothia aphylla* Rhynie Chert	Prostrate and aerial axes	Aseptate branched hyphae 10–15 μm in diam.	Globose thick‐walled, 80–150 μm	Absent	Globose to irregularly shaped up to 120 μm long and 75 μm wide	Rhizoids	Krings *et al*. (2007)
*Palaeoglomus boullardii*	*Horneophyton lignieri* Rhynie Chert	Aerial axis	Aseptate hyphae 5–6.5 μm in diam. Hyphae 3 μm attached to spores	Globose single bilayered, smooth Thin‐walled spores 45–55 μm Septa on the branching hypha Thickness of the wall: 1.5 μm	Present	Oval 30 μm × 40 μm to globose *c*. 50 μm	Likely through the epidermis	Strullu‐Derrien *et al*. (2014)

*Rugososporomyces lavoisierae* differs from the unnamed fungus described by Boullard & Lemoigne (1971) and fungus number 3 described by Krings *et al*. (2007) notably by the occurrence of arbuscules, which have not been observed in these two fungi.

*Rugososporomyces lavoisierae* can be distinguished from *Palaeoglomus boullardii* (Strullu‐Derrien *et al*., 2014) primarily on the structure of the spores. *Rugososporomyces lavoisierae* has spores with a thicker (3.1 μm), rugose wall and a septum on the subtending hypha, whereas those of *P. boullardii* are thinner (1.5 μm), smooth‐walled and have a subtending hypha that is continuous with the spore wall and closed by the invaginated inner layer of the spore wall. The ratio of spore to subtending hyphal diameter is higher in *P. boullardii* (15–18 : 1) and *G. rhyniensis* (10–18 : 1) than in *R. lavoisierae* (10 : 1).

We have not found reports of a similar fungus from deposits of this age and therefore describe it as novel here.

#### Comparison with extant mycorrhizas

The intracellular fungal colonization occurred in the aerial axis of *A. majus* which, like the other Rhynie chert plants, did not have roots. Structures formed by *R. lavoisierae* resemble the colonization by *Glomeromycotina* of extant thalloid liverworts, hornworts, lycophytes and ferns (Strullu‐Derrien *et al*., 2014; Field *et al*., 2015; Pressel *et al*., 2016; Rimington *et al*., 2018; Hoysted *et al*., 2019; Delaux & Schornack, 2021). Similarities include form, size and structure of the spore wall, H connections between parallel strands, intra‐axial vesicles and arbuscules. Furthermore, spores have a single hyphal attachment that persists, which is also a feature of extant *Glomus*. As Devonian fossil plants are evolutionary and structurally closer to extant bryophytes and lycophytes, we argued that comparisons with the latter rather than with gymnosperms and angiosperms are much more appropriate when interpreting the anatomy of early land plant–fungus symbioses (Strullu‐Derrien *et al*., 2014).

## Discussion

Three species of AM fungi showing clear evidence of arbuscules have now been documented in two species of plant from Rhynie (Taylor *et al*., 1995; Strullu‐Derrien *et al*., 2014; this paper). Our new species is the second species to be recorded in *Aglaophyton majus*. The first was *Glomites rhyniensis* (Taylor *et al*., 1995). These two records come from two different stratigraphic units within the chert beds at Rhynie. *G. rhyniensis* was described from the Rhynie Chert Unit, *R. lavoisierae* comes from the Windyfield Chert Unit. Fayers & Trewin (2004) noted in the caption of their fig. 6G: ‘endotrophic mycorrhizae in the cortex of a *Ventarura* rhizome’, but no description was provided, and the image of fungal spores is not informative enough to support the conclusion that mycorrhizas were present. *R. lavoisierae* is therefore the first documented AM fungus from the Windyfield Chert Unit. This shows that stratigraphically separate chert units at Rhynie supported the formation of AM. It is unlikely that the species diagnostic characters recognized result from different decompositional states of a single species. The characters are quite distinctive and carefully chosen, and each species has several distinctive features (see Table 1). The presence of different fungal species mirrors differences in plant and arthropod species, reflecting differences in the environments of the Rhynie and Windyfield units. Furthermore, the existence of multiple fungal species, as observed in extant plants, suggests that a varied fungal community may have been critical for the success and resilience of early terrestrial ecosystems.

The patterns of tissue colonization in *Aglaophyton majus* by *Glomites rhyniensis* (Taylor *et al*., 1995) and by *Rugososporomyces lavoisierae* are broadly similar. Both developed an extensive network of intercellular hyphae in the inner cortex of the aerial axis of the plant and a very distinctive zone of intracellular arbuscules in a narrow ring at the perimeter of the inner cortex. The colonization of the hypodermis (i.e. outer cortex) by *G. rhyniensis* differed from that of the inner cortex. In the hypodermis, the hyphae were thick‐walled and highly branched. Taylor *et al*. (1995) also documented large (25 μm diameter) ‘extraradical’ hyphae forming cord‐like structures. Neither hypodermal colonization nor extra‐axial hyphae were observed in *R. lavoisierae*. The comparison between *R. lavoisierae* and *G. rhyniensis* (above) shows that *A. majus* formed symbioses with at least two distinct species of AM fungi that had similar general patterns of tissue colonization but differed primarily in the size and structure of their spores and vesicles and in the dimensions of certain hyphae.

Living plant cells and fungal hyphae have distinctly different chemical compositions. Fungal hyphae are rich in chitin, whereas the plant water‐conducting cells imaged were predominantly composed of cellulose but probably also contained a lignin component. The original chemistry of the cell walls becomes altered during fossilization and diagenesis, but evidence from several sources indicates that taxon‐specific and even tissue‐specific signals persist in the organic remains of the organisms at Rhynie. High‐resolution analysis of tracheid cell walls of the fossil plant *Asteroxylon mackiei* using carbon X‐ray absorption near‐edge spectroscopy (C‐XANES) provided evidence of hydroxylated aromatic and aliphatic carbon in the inner and outer wall regions, which was interpreted as reflecting the distribution of lignin and structural polysaccharides (Boyce *et al*., 2002). The same method later showed that the conducting cells of *A. majus* and *Rhynia gwynne‐vaughanii* contained less aromatic carbon than those of *A. mackiei* (Boyce *et al*., 2003). The same oxygen‐bonded carbon (i.e. C–O, C═O and O–C═O) was detected in *R*. *gwynne‐vaughanii* using time‐of‐flight secondary ion mass spectrometry (ToF‐SIMS), but the aliphatic/aromatic ratio was not seen to vary greatly across tissue systems (Abbott *et al*., 2018).

The Raman spectra of our sample provide information about the organization of the aromatic skeleton, mostly about sp^2^ bonds (Ferrari & Robertson, 2000, 2001a, 2001b). The spectra show that the carbonaceous materials of the arbuscules and the vesicles of the water‐conducting cells are geologically altered by their diagenetic history and that they now have similar composition (Fig. 3a–h). The G and D peaks and subbands at *c*. 1266 and 1450 cm^−1^, along with their dispersive behaviors, indicate the presence of sp^2^ clusters *c*. 1.1 nm in size as well as carbon chains and C‐H bonds. The mean length of the sp^2^ clusters is in broad agreement with High‐Resolution Transmission Electron Microscopy results from Delarue *et al*. (2016) for another Rhynie Chert fossil, where the length scale of carbon clusters was found to be *c*. 0.55 nm. Loron *et al*. (2023) performed ATR‐FTIR studies on Rhynie chert microorganisms. They found absorption bands of organic matter in the interval 3000–2800 cm^−1^ and 1800–1400 cm^−1^ that they interpreted as characteristic absorptions for different CH, C=O, COOH and nitrogen moieties. They suggested that these organic groups represent the fossilization products of biomass that was originally dominated by lipids, proteins and sugars. They then transformed the intensity of informative organic bands (as revealed by the multivariate analyses) for each specimen (excluding the plant spores) into a matrix and conducted a discriminant analysis. By using unsupervised clustering methods (principal components analysis and K‐means), the authors considered that the observed differences reflect variations in the original precursors of the fossil organic matter, modified through diagenesis. Our results are consistent with another study of Rhynie chert carbonaceous material that used Raman spectrometry, which indicated that the carbonaceous material has experienced advanced diagenesis (Qu *et al*., 2015).

In our specimens, the residual carbonaceous materials of the fungus and the vesicles in the plant cell walls return a similar very short (0.3 ns) lifetime fluorescence. These structures are also the most resilient ones within the plant axis, and they have a similar brown color under brightfield illumination. Our explanation for the similar fluorescence lifetimes is that the chitin in the fungal arbuscules and the lignocellulose vesicles in the water‐conducting cells have decomposed to simpler but similar chemical breakdown products, as shown by the Raman analyses. This result can be compared to those obtained on the earliest known wood (from coeval deposits to the Rhynie Chert), which was anatomically exceptionally preserved. Transmission X‐ray Microscopy‐based X‐ray Absorption Near Edge Structure (TXM–XANES) and Transmission electron microscopy analyses performed on specimens preserved in 2D and 3D also showed that the fossil wood was geologically altered by its diagenetic history. The remaining organic matter, in both specimens, had a similar chemical composition that does not reflect the original composition of the wood, even if a lignin source for the original compounds cannot be ruled out completely (Strullu‐Derrien *et al*., 2019).

In conclusion, the integration of brightfield microscopy, advanced confocal‐FLIM imaging and Raman analyses enabled the identification of a 407‐Myr‐old arbuscular mycorrhizal symbiosis in the Windyfield Chert. The exquisite preservation of the fossils, combined with advanced imaging, revealed carbonized fungal structures in unprecedented detail, supporting the re‐examination of other fossil specimens using confocal‐FLIM analyses. The Raman analyses further demonstrated that anatomical and chemical preservation are not necessarily correlated, consistent with previous findings. Future work should assess how widespread *Rugososporomyces lavoisierae* is within the Rhynie and Windyfield cherts, determine the extent of its colonization of different tissues (e.g. rhizoids) and host plants, and explore the broader diversity of symbiotic associations preserved in the Windyfield Chert. Such studies will provide new insights into the complexity of early plant–fungal interactions in the fossil record.

## Competing interests

None declared.

## Author contributions

CS‐D and SS conceptualized the study. CS‐D, SS and GE collected the data in brightfield microscopy. CS‐D, FF, SS, RW and GE collected the confocal data; RW collected the FLIM data. LPM and ACF performed the Raman analyses. All the authors analyzed their data. CS‐D and PK wrote the original draft with the input of RW, SS, LPM and ACF. All authors contributed to the final version of the manuscript. RW and LPM contributed equally to this work.

## Disclaimer

The New Phytologist Foundation remains neutral with regard to jurisdictional claims in maps and in any institutional affiliations.

## Supporting information


**Fig. S1** Overlay of a Raman map acquistion showing intensity of the G‐band peak at 1602 cm^−1^ on a brightfield image of the stem.
**Fig. S2** Vesicles in the vascular strand.
**Notes S1** Identification of *Aglaophyton majus* – the plant hosting the arbuscules.

**Video S1** Separation of structural features of fungal arbuscules and hyphae based upon fluorescence lifetime.
Please note: Wiley is not responsible for the content or functionality of any Supporting Information supplied by the authors. Any queries (other than missing material) should be directed to the *New Phytologist* Central Office.

## Data Availability

All confocal data collected and used in this study are deposited in the Zenodo repository under a Creative Commons Attribution 4.0 international license https://doi.org/10.5281/zenodo.15194427 (Strullu‐Derrien *et al*., 2025). Accession number: slide no. NMS G.2022.11.48.1 is housed at the National Museum of Scotland, Edinburgh. Yves Candela (y.candela@nms.ac.uk) can be contacted for access.
