# Supplementary material for: An arbuscular mycorrhiza from the 407‐million‐year‐old Windyfield Chert identified through advanced fluorescence and Raman imaging
Source: New Phytol. 2025 Nov 12;249(1):448–59. doi: 10.1111/nph.70655 (PMC12676081; doi:10.1111/nph.70655)
Supplement: Supplementary file 1 — Fig. S1 Overlay of a Raman map acquistion showing intensity of the G‐band peak at 1602 cm−1 on a brightfield image of the stem. Fig. S2 Vesicles in the vascular strand. Notes S1 Identification of Aglaophyton majus – the plant hosting the arbuscules. [file NPH-249-448-s001.pdf]

**New phytologist Supporting Information**

# An arbuscular mycorrhiza from the 407-million-year-old Windyfield chert identified through advanced fluorescence and Raman imaging

Christine Strullu-Derrien, Raymond Wightman, Liam Patrick McDonnell, Gareth Evans, Frédéric Fercoq, Paul Kenrick, Andrea C. Ferrari, Sebastian Schornack

Article acceptance date: 25 September 2025

Multiple Raman spectra ROIs were chosen based on the fluorescence lifetime imaging

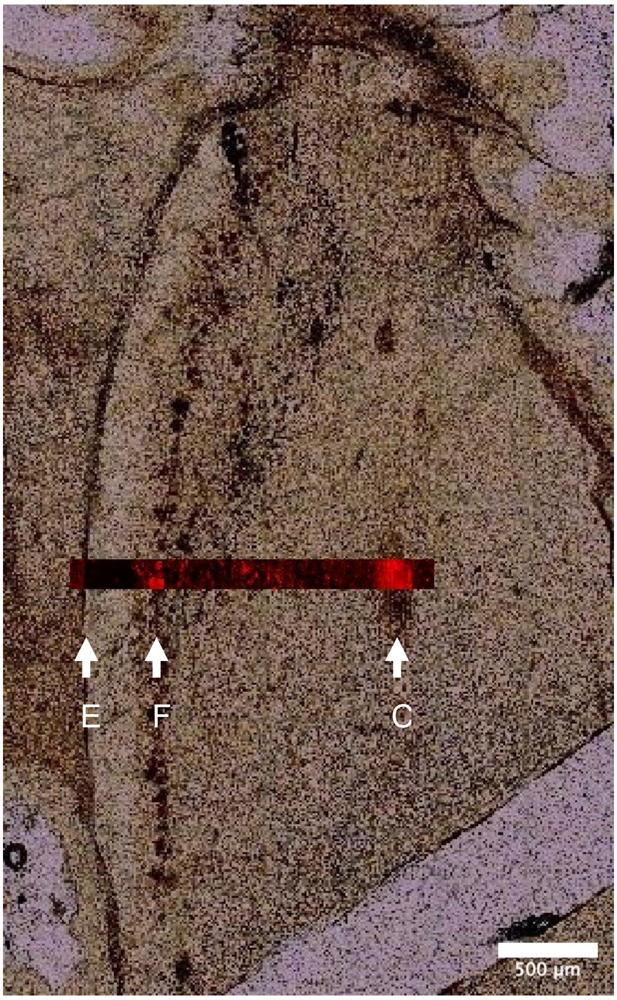


Figure S1. Overlay of a Raman map acquistion showing intensity of the G-band peak at 1602 $cm^{-1}$ (red pixels) on a brightfield image of the stem. The Raman map transects the stem from the epidermis (E) through the fungal zone (F) to the conducting tissue (C). Between points E and F, which is devoid of fungal structures, G-band peaks are not observed.

**Notes S1**. Identification of *Aglaophyton majus* – the plant hosting the arbuscules

The identity of the large plant containing the arbuscular fungus was determined as *Aglaophyton majus* based on observations of axes in two consecutive thin sections made from the same block of chert (slides no. NMS G.2022.11.48.1 and no. NMS G.2022.11.48.2 at the National Museum of Scotland, Edinburgh).

The fungal arbuscules imaged with the confocal microscope are inside an axis in slide RW 14.1. The outlines of epidermal cells and two stomata are preserved in the cuticle. Few other plant cells are clearly distinguishable. At low magnification, there is a distinctive zone of brown bodies; this is the fungal arbuscular zone. These brown-coloured bodies of low opacity measure~60–80 µm in diameter. The arbuscules are well identified by confocal and FLIM microscopy. They colonized turgescent cells (Fig. S1A) meaning that the plant was alive at the time of their formation.

The most distinctive tissue system that remains is a part of the vascular system in the centre of the axis. This is a cylinder of cells which corresponds to the inner zone of thin-walled water-conducting cells. Further confirmation of this identity comes from high-resolution imaging with the confocal microscope (Fig. S1B). These cells contain numerous vesicles of varying size that are typically associated with a thin underlying cell wall. The vesicles are organised into chains that can partly occlude the cell lumen. The perimeter of the vesicles is somewhat angular. These features are diagnostic of the water-conducting cells of *A. majus* (see Remy & Hass, 1996; Plate IV fig. 5). Their origin and function are poorly understood. They have been variously interpreted, and more recently as part of the original cell wall structure (Remy & Hass, 1996; Edwards, 2003; Kerp, 2018). It is the first time that these vesicles have been observed in 3D.

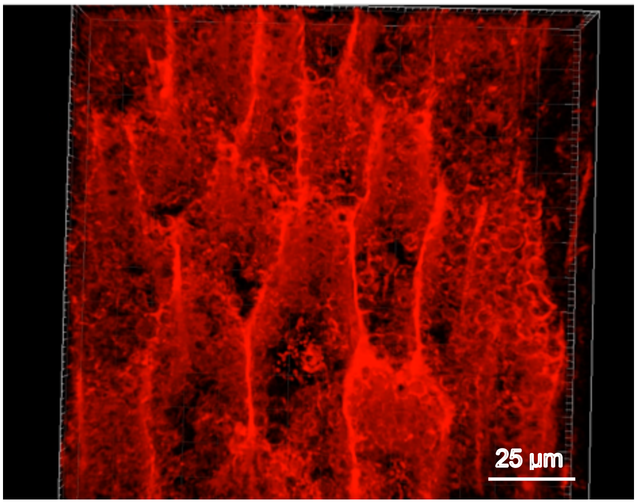


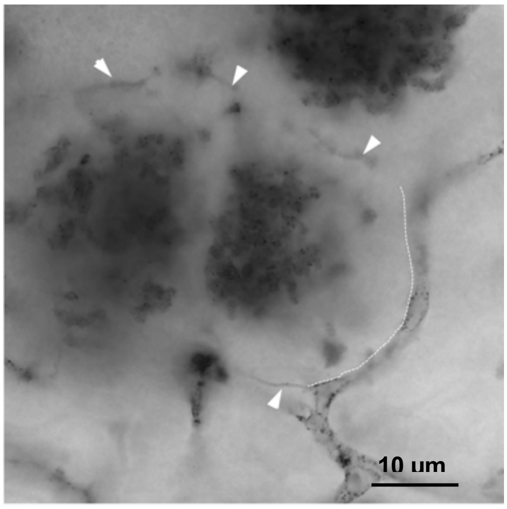

Figure S2. Vesicles in the vascular strand. A. 3D projection; B. Arbuscules in cells, arrowheads and dashed line depict the cell walls.

Edwards D. 2003**.** Xylem in early tracheophytes. *Plant Cell and Environment* **26**(1): 57–72.
Kerp H. 2018. Organs and tissues of Rhynie chert plants. *Philosophical Transactions of the Royal Society B: Biological Sciences* **373**(1739): 20160495.
